# Prediction of metachronous multiple primary cancers following the curative resection of gastric cancer

**DOI:** 10.1186/1471-2407-13-394

**Published:** 2013-08-23

**Authors:** Chan Kim, Hong Jae Chon, Beodeul Kang, Kiyeol Kim, Hei-Cheul Jeung, Hyun Cheol Chung, Sung Hoon Noh, Sun Young Rha

**Affiliations:** 1 Yonsei Cancer Center, Yonsei University College of Medicine, Seoul, Korea; 2 Department of Internal Medicine, Yonsei University College of Medicine, Yonsei-ro, Seodaemun-Ku, 120-752, Seoul, Korea; 3 Yonsei Cancer Research Institute, Seoul, Korea; 4 Oral Cancer Research Institute, Yonsei University Health System, Seoul, Korea; 5 Brain Korea 21 Project for Medical Science, Yonsei University College of Medicine, Seoul, Korea; 6 Department of Surgery, Yonsei University College of Medicine, Seoul, Korea

**Keywords:** Multiple primary cancer, Gastric cancer, Nomogram, Predictive factor

## Abstract

**Background:**

Due to improved survival rate, gastric cancer (GC) patients have an increased risk of developing multiple primary cancer (MPC). The purpose of this study is to evaluate the clinicopathological features of MPC and to generate useful tools for the prediction of metachronous MPC following gastrectomy.

**Methods:**

3066 patients who underwent curative resection of GC were reviewed retrospectively, based on the clinical information and the medical record.

**Results:**

The 5-year incidence of MPC was 2.5%. Of these, 54.3% had a metachronous MPC, while 45.7% had a synchronous MPC. The most prevalent site of metachronous MPC was the colorectum (26.3%), followed by lung (23.7%) and liver (18.4%). Multivariate logistic regression analysis revealed that old age at the time of GC diagnosis (≥60 years), early stage of GC (stage I and II), and multiplicity of GC at the time of gastrectomy were independent predictive factors for metachronous MPC. GC patients with either metachronous or synchronous MPC showed poorer survival than patients without MPC. In addition, patients with a metachronous MPC showed late survival disadvantage, while patients with a synchronous MPC showed early survival disadvantage. Furthermore, we were able to develop and internally validate a nomogram to predict the metachronous MPC after curative gastrectomy (C-index = 0.72).

**Conclusion:**

Patients at high risk of developing metachronous MPC after curative resection of GC were identified. Individual risk of developing metachronous MPC could be predicted by a novel nomogram. Further external validation with independent patient cohorts is required to improve the accuracy of prediction.

## Background

Gastric cancer (GC) is the fourth most common cancer and the second leading cause of cancer-related death worldwide, and is especially prevalent in the Asia-Pacific region [1]. In recent years, the emphasis on regular cancer screening programs and advances in diagnostic techniques has greatly improved the detection rate of early gastric cancer (EGC) [2]. In Korea, a population-based mass-screening program for GC was initiated by the Ministry of Health and Welfare in 2002. This screening program recommends a biennial upper gastrointestinal series or endoscopy for people over 40 years old [3]. Although this screening program does not completely cover the target population, it already has led to an increase in the detection rate of EGCs from 33% in 1999 to 60% in 2012, and has subsequently contributed to an improved clinical outcome for GC [4]. In addition, advances in surgical techniques and multimodal treatments have also improved survival [2,5,6]. Because of this prolongation in survival, GC patients live longer, but have a greater possibility of developing multiple primary cancer (MPC).

Since MPC could influence the overall prognosis of GC, early screening and proper management of MPC in GC survivors is of particular importance. To date, few studies have investigated the incidence and clinical pattern of MPC in GC patients and most of these studies have been limited to some subsets of patients. Moreover, little is known about the risk factors predictive of MPC following curative gastrectomy [7-11].

The aim of the present study was to determine the clinicopathological features and outcomes of MPC, with the goal of generating useful predictive tools for MPC in GC survivors.

## Methods

Between 2000 and 2004, 3066 patients underwent resection of GC with the curative aim at the Yonsei Cancer Center, Severance Hospital (Yonsei University Health System, Korea). All of these patients were analyzed retrospectively using the medical record from our institute. The study was approved by the institutional review board of Severance Hospital. The criteria of Warren and Gates [12] were used to define MPC: 1) The tumor must have definite malignancy features; 2) The tumor has to be separate and distinct from the index tumor, which was gastric adenocarcinoma in the present study; 3) The possibility of the tumor being a metastasis of the index tumor should be ruled out. Patients with multiple primary GC in the remnant stomach after gastrectomy were not included.

All patients underwent a complete blood count, routine chemistry, upper endoscopy, chest radiography, and abdominal computed tomography at the time of GC diagnosis. Clinicopathological characteristics, including sex, age of GC diagnosis, stage, multiplicity, location, cell type of GC, type of gastrectomy, maximal length of GC mass, initial CEA level, CA 19–9 level, site of MPC, time interval between GC and MPC, history of smoking and alcohol, and clinical outcome were assessed. Multiple gastric cancer included multiple synchronous gastric cancers, multiple cancers in the remnant stomach, and the GC patients with the history of prior endoscopic mucosal resection (EMR) or endoscopic submucosal dissection (ESD).

Pathological diagnosis and classification of the cancer was made according to the criteria of the American Joint Committee on Cancer 2002 staging system. For the diagnosis of hepatocellular carcinoma, the AASLD (American Association for the Study of Liver Disease) criteria were used, which consist of elevated serum α-fetoprotein level > 200 ng/ml or typical pattern of enhancement on dynamic imaging of hepatic mass . 2 cm in a cirrhotic liver [13]. After being discharged from the hospital, all patients took part in a regular follow-up program. Patients were followed-up on every three months within the first two years, every four months during the third year, every six months during the fourth and fifth year, and once every year thereafter. Synchronous MPC was defined as MPC diagnosed within 6 months of GC diagnosis, while metachronous MPC was defined as MPC diagnosed more than 6 months after the GC diagnosis. Overall survival was defined as the time from the curative gastrectomy to death or to last follow-up. We reviewed the causes of death based on the medical records of our institute and the survival database of the National Statistical Office.

The Statistical Package for Social Sciences version 17.0 for Windows (SPSS, Inc., Chicago, IL) was used for statistical analysis. Chi-square tests and independent sample t-test were used for the analysis of variables. The survival curve was evaluated using the Kaplan-Meier method, and statistical differences were analyzed using the log-rank test. The accepted level of significance was p < 0.05.

The nomogram was established by using selected risk factors for predicting an individual patient’s probability of developing metachronous MPC within 5-years after gastrectomy. For the development of MPC predicting nomogram, 32 patients with synchronous MPC were excluded, and remaining 3034 patients were analyzed following Kattan’s method [14]. The nomogram was validated using concordance index (c-index) and a calibration plot [15]. Calibration was performed by comparing the accuracy between the actual incidence of MPC and the nomogram-predicted incidence of MPC. The statistical analysis for the nomogram was performed using R program (http://www.r-project.org/).

## Results

### Patient characteristics and detection of MPC

Table 1 shows the baseline characteristics of the cohort. The mean age at diagnosis of GC was 57.3 years old and the male-to-female ratio was 2.06 to 1. The median follow-up duration was 60.3 months. 587 patients (18.9%) experienced the recurrence after the curative resection of GC. Among recurred patients, most common location of recurrence was the peritoneum (162 cases), followed by lymph node (100 cases), liver (88 cases), anastomosis site (39 cases), bone (22 cases), and remnant stomach (20 cases).

**Table 1:** Baseline characteristics of GC

	**Frequency (%)**
	**(*n*** **= 3066)**
**Sex**	
Male	2065 (67.4%)
Female	1001 (32.6%)
**Age at diagnosis**	57.3 ± 11.8
**Stage**	
I	1640 (53.5%)
II	448 (14.6%)
III	736 (24.0%)
IV	242 (7.9%)
**Multiplicity**	
Single	2860 (93.3%)
≥2	206 (6.7%)
**Location**	
Upper	1516 (50.4%)
Mid	1023 (34.0%)
Lower	351 (11.7%)
Diffuse	116 (3.9%)
**Cell type**	
Tubular	2390 (78.0%)
Adeno WD	406 (13.2%)
Adeno MD	859 (28.0%)
Adeno PD	1125 (36.7%)
Signet ring cell	514 (16.8%)
Mucinous	73 (2.4%)
Others	89 (2.9%)

Among 3,066 patients with GC, 70 (2.3%) were found to have a MPC. Of these, 38 (54.3%) had a metachronous MPC, while 32 (45.7%) had a synchronous MPC. When considering the censored cases, the 5-year cumulative incidence of metachronous MPC after the diagnosis of GC was 1.4%. The majority of metachronous MPC occurred within three years from the diagnosis of GC. The mean interval between the diagnosis of GC and metachronous MPC was 25.5 months. However, some metachronous MPCs were found even after three years, suggesting that metachronous MPC can occur at any time after gastrectomy. The 5-year mortality rate was 23.6% for patients without MPC, while the 5-year mortality rate was 65.8% for patients with metachronous MPC.

The most common site of metachronous MPCs among GC patients was colorectum (10 cases, 26.3%), followed by lung (9 cases, 23.7%), liver (7 cases, 18.4%), gallbladder (5 cases, 13.2%), and head and neck (4 cases, 10.5%). In addition, Of 38 patients with metachronous MPC, 34.2% were at stage I, 44.7% at stage II, and 21.1% were at stage III.

### Predictive factors for MPC

Table 2 shows a clinicopathologic comparison between the patients with and without metachronous MPC. The metachronous MPC group had more male (84.2% vs. 67.1%, *P* < 0.025) and older (63.6 ± 7.7 vs. 57.2 ± 11.8, *P* < 0.001) patients than the group without MPC. In addition, the stage of GC was earlier in patients with metachronous MPC than in patients without MPC (*P* = 0.011). No stage IV GC patients developed the metachronous MPC probably due to relatively short survival (median survival: 10.8 months). In addition, multiple GCs at the time of gastrectomy were more common among patients with metachronous MPC (23.7% vs. 6. 5%, *P* < 0.001). There were more patients with the history of alcoholic drinking in metachronous MPC group (68.4% vs. 48.4%, *P* = 0.048). No differences were found in the location, cell type of GC, initial CEA level, initial CA 19–9 level, and smoking history between two groups.

**Table 2:** Clinicopathological characteristics of GC according to presence of metachronous MPC

	**No MPC**	**Meta MPC**	***P*value**
	**(*n*** **= 2996)**	**(*n*** **= 38)**	
**Sex**			
Male	2010 (67.1%)	32 (84.2%)	0.025
Female	986 (32.9%)	6 (15.8%)	
**Age**	57.2 ± 11.8	63.6 ± 7.7	<0.001
**Stage**			
I	1598 (53.3%)	26 (68.4%)	0.011
II	430 (14.4%)	9 (23.7%)	
III	726 (24.2%)	3 (7.9%)	
IV	242 (8.1%)	0 (0%)	
**Multiplicity**			
Single	2802 (93.5%)	29 (76.3%)	<0.001
≥2	194 (6.5%)	9 (23.7%)	
**Location**			
Upper	1481 (50.4%)	19 (52.8%)	0.709
Mid	1003 (34.1%)	10 (27.8%)	
Lower	339 (11.5%)	6 (16.7%)	
Diffuse	115 (3.9%)	1 (2.8%)	
**Cell type**			
Tubular	2334 (77.9%)	32 (84.2%)	0.703
Signet ring cell	503 (16.8%)	5 (13.2%)	
Mucinous	72 (2.4%)	0 (0%)	
Others	87 (2.9%)	1 (2.6%)	
**Maximal tumor size**	4.1 ± 2.8	3.0 ± 1.8	<0.001
**Initial CEA**	4.6 ± 21.5	3.4 ± 4.1	0.763
**Initial CA 19-9**	40.1 ± 319.1	8.0 ± 6.9	0.662
**Smoking History**			
Current smoker	804 (27.2%)	14 (36.8%)	0.402
Ex-smoker	406 (13.7%)	4 (10.5%)	
Never-smoker	1746 (59.1%)	20 (52.6%)	
**Alcoholic History**			
Current drinker	1235 (41.8%)	22 (57.9%)	0.048
Ex-drinker	196 (6.6%)	4 (10.5%)	
Never-drinker	1521 (51.5%)	12 (31.6%)	
**Outcome**			
Alive	2290 (76.4%)	13 (34.2%)	
Dead	706 (23.6%)	25 (65.8%)	

Multivariate logistic regression analyses were performed for variables that showed significance in the univariate analysis. For metachronous MPC cases, age older than 60 years at the time of GC diagnosis, early stage (I, II) of GC, and multiplicity of GC were independent predictive factors (Table 3).

**Table 3:** Logistic regression analyses of risk factors for metachronous MPC

	**Metachronous MPC**	
**Variables**	**RR (95% CI)**	***P*value**
**Sex** (female vs male)	1.51 (0.54-4.21)	0.436
**Age** (<60 vs ≥60)	2.46 (1.20-5.07)	0.014
**Stage** (III, IV vs I, II)	4.82 (1.37-16.97)	0.014
**Multiplicity** (Single vs Multiple)	6.76 (3.05-14.96)	<0.001
**Maximal length** (≥3.5 cm vs <3.5 cm)	1.35 (0.64-2.84)	0.428
**Alcohol history** (No vs Yes)	1.87 (0.83-4.22)	0.131

### MPC and survival disadvantages

The 5-year survival rate for all patients was 75.9%. This high survival rate seemed to be caused by a relatively high proportion of earlier stage GC (stage I cancer: 53.5%, stage II: 14.6%), which is the result of the nationwide screening program for GC in Korea.

**Figure 1:**
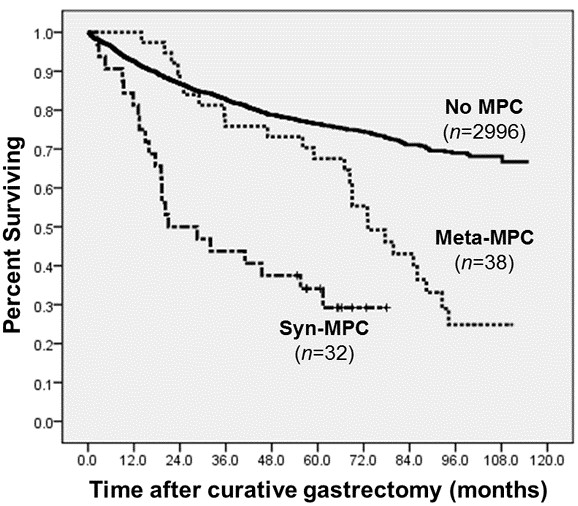
**MPC and survival disadvantages in GC patients.** The Kaplan-Meyer survival curves according to the type of MPC. P < 0.001 for Meta-MPC vs. No MPC, p < 0.001 for Syn-MPC vs. No MPC.

The 5-year survival rate was 76.5% for patients without MPC, 67.5% for patients with metachronous MPC, and 34.1% for patients with synchronous MPC (Figure 1). Comparison of the survival curves of the metachronous and synchronous MPC patients to that of patients without MPC showed that both metachronous MPC and synchronous MPC patients had poorer survival than patients without MPC (*P* < 0.001 and *P* < 0.001, respectively). In addition, analysis of patients by the stage of GC showed that the survival of patients with metachronous MPC was poorer than that of patients without MPC for all stages (*P* < 0.001 for stage I, *P* = 0.004 for stage II, and *P* = 0.039 for stage III).

Patients with metachronous and synchronous MPC showed different patterns of cause of death. Among 38 metachronous MPC patients, 25 (65.8%) died during follow-up, in which the major cause of death was the progression of MPC. In contrast, of 32 synchronous MPC patients, 22 (68.8%) died during follow-up, in which the major cause of death was the progression of both GC and MPC.

### Nomogram

**Figure 2:**
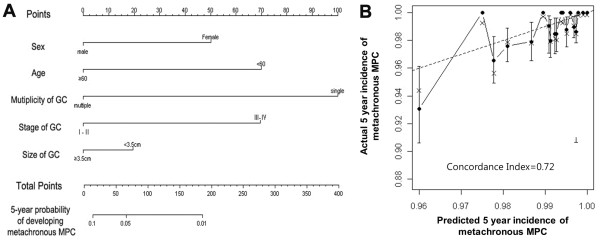
**Nomogram for prediction of 5-year probability of developing metachronous MPC. (A)** Nomogram for the prediction of metachronous MPC. Find the value of each variables on the variable axis and draw a vertical line upward to the ‘Points’ axis and determine the corresponding points for the variable. Sum the points achieved for each variables and locate this sum on the ‘Total Points’ axis. Draw a vertical line down to the 5-year probability axis to determine the patient’s probability of developing metachronous MPC within 5 years of gastrectomy. **(B)** Calibration plot for 5-year nomogram prediction. An ideal nomogram (dashed line) and the current nomogram (solid line). Vertical bars indicate 95% confidence intervals.

For prediction of developing metachronous MPC, we tried to generate a nomogram based on Cox regression. From the Cox regression model, male (p = 0.029), age over 60 years (p = 0.002), multiplicity of GC (p < 0.001), and earlier stage (p = 0.026) were associated with the development of metachronous MPC. We tried to improve the c-index by several strategies such as combining and adding various variables and finally generated the nomogram from 5 parameters that were determined at the time of gastrectomy (Figure 2A). The concordance index of the model was 0.72. Figure 2B illustrates the calibration plot of the nomogram. The practical application of this nomogram is as follows: one patient got 0 point for his sex (male), 0 point for his age (≥60), 0 point for multiplicity of GC (multiple gastric lesions), 0 point for stage of GC (stage II), and 20 point for tumor size (<3.5 cm), giving a total of 20 points. By relating the axes of total points and the probability of MPC, we are able to predict his probability of developing metachronous MPC in 5-year is about 9%.

## Discussion

Advances in diagnostic techniques and new chemotherapeutic drugs have improved the clinical outcome of cancer, and more cancer patients are surviving longer after their initial diagnosis [16]. In general, improved survival is suggested to be associated with an increased risk of developing MPC [17].

Although the major reason for the increased prevalence of MPC is increased survival, and therefore an extended risk period, other possible explanations have been proposed. First, genetic vulnerability associated with specific genes may play a role in the development of MPC. For example, hereditary nonpolyposis colorectal cancer, which is a syndrome associated with mutations in a class of genes such as MLH1, MSH2, and MSH6, is characterized by an increased susceptibility to other malignancies, especially of the uterus, ovary, urinary tract, and stomach [18,19]. Second, some carcinogenic environmental factors may induce multiple neoplasms of independent organs that were exposed to same carcinogens. The field cancerization effect, which is associated with an increased risk of multiple cancers in aerodigestive organs after prolonged exposure to cigarette smoking, is a well-known example of this phenomenon [20]. Lastly, modalities used in the treatment of the index cancer may also induce secondary cancers. For example, a relationship has been reported between combined therapy with radiotherapy and alkylating agents and an increased risk of gastric and colon cancer in survivors of Hodgkin’s lymphoma [21,22].

In GC, which is the fourth most common cancer in the world, the trend toward increasing MPC is similar. In the present study, of the 3066 GC patients who underwent curative gastrectomy, 70 patients (2.3%) had been diagnosed with a metachronous or synchronous MPC, which is consistent with results of previous reports that reported a range from 2.0 to 7.6% [7-9,11,23]. Few previous studies have reported the incidence and clinical pattern of MPC in GC patients who underwent curative gastrectomy. The majority of studies regarding MPC in GC have been performed in Eastern countries, with fewer studies performed in Western countries. Eom et al. reported that, in Korea, the most common MPC is colorectal cancer (20.8%), followed by lung cancer (11.9%) and liver cancer (11.3%) [7]. Ikeda et al. similarly reported that, in Japan, the most common MPC is colorectal cancer (32.6%), followed by lung cancer (28.4%) and liver cancer (8.4%) [8]. Meanwhile, Lundegardh et al. reported that, in Sweden, the most common site is the colorectum (19.9%), followed by lung (6.1%) and kidney (5.3%) [23]. In the present study, the most common site of metachronous MPC was the colorectum (26.3%), followed by lung (23.7%) and liver (18.4%), which were similar to other Asian report.

In the present study, the authors were able to identify predictive factors for metachronous MPC. The logistic regression analysis revealed that age over 60 years at the time of GC diagnosis, earlier stage GC (stage I, II), and multiplicity of GC at the time of gastrectomy were independent predictive factors for developing metachronous MPCs. When GC patients had multiple GC lesions at the time of gastrectomy, the 5-year cumulative incidence of MPC during follow-up was as high as 10.4%.

The survival analysis of this study showed that GC survivors with MPC had a remarkably poor survival when compared to patients without MPC. This survival disadvantage was most prominent for patients with early stage GC. Because the incidence of MPC was highest in early stage GC patients, they are possible candidates for MPC screening. In addition, patients with synchronous MPC showed the worst survival rate, and most death events occurred within the first 2 years following the gastrectomy. For metachronous MPC cases, however, the survival rate was fairly similar to that of patients without MPC until 5 years after gastrectomy, but after that time, the survival rate decreased rapidly. That is, metachronous MPC showed a late survival disadvantage, while synchronous MPC showed an early survival disadvantage. One possible explanation is that synchronous cancers may have negative influences on the general medical condition of the patient, thus hindering suitable therapeutic strategies for treatment of GC. In contrast, metachronous MPC develop many years after the treatment of GC and it does not change the treatment of GC in itself.

Another important point of the present study is that we were able to develop and internally validate a predictive nomogram for 5-year probability of developing metachronous MPC in GC patients following the curative gastrectomy. The c-index of this nomogram is 0.72, which means that its predictive accuracy is 72%. Though this nomogram could have been biased due to insufficient MPC event, to our knowledge, this is the first nomogram to predict metachronous MPC in GC patients. We fully expect that this nomogram could provide a more personalized prediction of MPC than independent risk factor models. Because the presented incidence of MPCs was so small, it may have biased the overall accuracy of the nomogram. Although the nomogram is not perfectly precise, our results provide better prediction of MPCs compared to other method. Further external validation with other independent group of patients is desirable to overcome this weakness and to improve the accuracy of our nomogram.

The small incidence of MPC in GC survivors was the major limitation of this study. In the present study, we applied very strict criteria to diagnose MPCs, which might be the cause of small incidence of MPCs. Because our study is fundamentally a retrospective study, it was hard to acquire the satisfactory patient data; we excluded as many as 27 ambiguous cases in which the distinction between MPCs and metastasis was not clear. In addition, the duration of the median follow-up (60.3 months) is not sufficient for the development of MPCs. Because of these limitations, this study showed relatively low incidence of MPCs. In reality, the incidence of MPC is not very rare. According to the National Cancer Institute’s SEER (Surveillance, Epidemiology, and End Results) data, in which the data collection was between 1973 and 2003, the incidence of MPC was approximately 9% [17]. The risk varies according to the type of primary index tumor ranging from 1% for primary liver cancer to 16% for bladder cancer. Therefore, the screening program for MPC may have a beneficial role. If we extend the duration of follow-up in future study, it will better characterize the predictive factors of MPCs. To date, there are several reports regarding the development of MPCs in GC survivors [7-9,23]. However, these are not sufficient to justify the routine screening for the detection of MPC in all GC survivors; it is also difficult to address the optimal screening protocol. Despite these considerations, because the incidence of MPC was not very rare and it negatively affected the clinical outcome of GC survivors in this and previous studies, we need to consider the possibility of MPCs, especially in the lesions where MPCs occur frequently. In particular, because this study showed that 63% of metachronous MPC developed within the abdominal cavity and 76% of metachronous MPCs developed within 3 years after the gastrectomy, we consider it necessary for clinicians to pay particular attention to the follow-up abdominal imaging of GC survivors for at least 3 years after GC diagnosis. We assume that there will be, in fact, more cases of overlooked MPCs by misdiagnosis as metastases of GCs. Aggressive tissue biopsy can be helpful in differential diagnosis, and can play a decisive role in determining proper treatment strategies in GC patients with predictive factors such as old age at the time of diagnosis (≥60 years), early stage (I, II), and multiplicity of GCs.

## Conclusion

In conclusion, in this study, we could analyze the incidence and clinical pattern of MPC following the curative gastrectomy in GC patients. In particular, we could unearth that factors such as old age at the time of GC diagnosis (≥60), earlier stage GC (stage I, II), and multiple GC are valuable predictive factor for MPC. Furthermore, we have generated and validated a nomogram for predicting individual probability of developing metachronous MPC. If we could screen the development of metachronous MPC by using these useful tools, we could improve the clinical outcome of GC survivors.

## Abbreviations

GC: Gastric cancer; MPC: Multiple primary cancer; EGC: Early gastric cancer; WD: Well differentiated; MD: Moderately differentiated; PD: Poorly differentiated.

## Competing interests

All authors disclosed no potential conflicts of interests.

## Authors’ contributions

CK, KK, HCJ, HCC, SHN, SYR have made substantial contributions to conception and design of the study. CK, BK carried out acquisition of data. CK, KK, HCC, SHN, SYR carried out analysis and interpretation of data. CK, KK, SYR have been involved in drafting the manuscript. All authors read and approved the final manuscript.

## Pre-publication history

The pre-publication history for this paper can be accessed here:

http://www.biomedcentral.com/1471-2407/13/394/prepub
